# Ensuring justice in global health education initiatives – a review of pearls and pitfalls

**DOI:** 10.3389/fpubh.2025.1574917

**Published:** 2025-05-27

**Authors:** Aleksandar Stevanović, Vesna Bjegovic-Mikanovic, Ulrich Laaser

**Affiliations:** ^1^Institute of Social Medicine, Faculty of Medicine, University of Belgrade, Belgrade, Serbia; ^2^Faculty of Health Sciences, Bielefeld University, Bielefeld, Germany

**Keywords:** global health, health education, health equity, health workforce, international cooperation

## Abstract

Global health education initiatives (GHEIs) include various educational programs and opportunities aimed at training a competent global and public health workforce to address major global health issues. Many GHEIs are led by institutions in high-income countries and fail to support the development of local educational capacities that would strengthen health systems and ensure a continuous supply of public health professionals. This analysis explores the barriers to equitable GHEIs, emphasizing the importance of justice-related aspects. Despite substantial investments in GHEIs in recent years, there is a risk of reinforcing existing inequities due to systemic injustice and power dynamics. We identify six key areas for improvement regarding these barriers: socioeconomic equity, gender equity, inclusiveness and disability justice, decolonization principles, sustainability and transparency, and the environmental footprint in education. We argue how enhancing each of these aspects can break down existing injustice and enable disadvantaged individuals to fully participate in GHEIs. Furthermore, we propose a set of indicators to identify and address common barriers during the design, implementation, and evaluation of GHEIs. Promoting equitable access, meaningful representation, and sustainable collaboration in GHEIs is an important step toward achieving global health justice and reducing dependence on foreign support.

## 1 Introduction

A high-quality training process for new public health professionals (PHPs) is necessary for all countries to ensure a competent workforce, implement public health interventions, and promote sustainable development (1). Public health expertise strengthens health systems and equips countries to respond effectively to health crises and reduce the burden of disease. However, the field of public health continuously grows more complex, and the responsibilities of PHPs are also expanding (2). Today’s public health workforce is expected to tackle traditional public health challenges and address complex issues such as climate change, antimicrobial resistance, and the declining public trust in health systems and science (3). This work should be done through multidisciplinary efforts coordinated by PHPs equipped with the knowledge and skills required to answer essential public health operations. A steady supply of such competent PHPs is critical for each country to build resilient, adaptive public health systems capable of responding to emerging global health threats (4).

Despite the growing need for competent PHPs worldwide, the opportunities for advanced public health training are disproportionately concentrated in high-income countries (HICs) (5) due to the historical injustice that has led to an unequal distribution of educational resources, scientific influence and economic power. Today’s leading public health education institutions are located in HICs, where they set professional standards and advance public health research. While many of these institutions have expanded their global health programs to address public health crises in low- and middle-income countries (LMICs), they fail to support the development of local educational capacities that would enable LMICs to have a continuous local supply of competent PHPs (6, 7). Instead, these programs focus on attracting students to high-income countries, where the curriculum, research priorities, and standards are shaped by wealthier nations’ health needs and realities (5). This creates a gap between the educational outcomes and the actual needs of LMICs (5, 8–10).

Although many national and international bodies concerned with global and public health (as *host institutions*) have recognized the importance of inclusivity and accessibility of global health education initiatives (GHEIs), their actions to reach these standards have been limited (11, 12). Growing awareness of the importance of sustainability in education and the colonial legacy of many host institutions has led to the expansion of GHEIs for students from LMICs. GHEIs usually include short-term courses, summer school programs, workshops, and master’s programs in global and public health. GHEIs are often promoted as accessible and inclusive and may include benefits like reduced tuition fees, scholarships, and financial support for travel or accommodation. Although these actions can present a positive step, they often fail to create fully equitable opportunities (13). We argue that many hidden barriers to enrolling in such GHEIs remain and can prevent qualified and motivated candidates from accessing them (10, 14, 15).

A more systematic approach is required to address LMIC students’ barriers and ensure a more equitable distribution of public health knowledge and expertise (10, 13, 15). Host institutions must consider a set of standardized equity indicators when assessing the accessibility, inclusiveness, sustainability, and impact of their GHEIs. Moreover, these initiatives should aim to strengthen local educational capacities if the overarching goal is to decrease dependence on foreign support and tackle the power imbalance (15). Defining an objective set of indicators could help determine which initiatives support LMICs in training PHPs with the skills to tackle their countries’ specific health challenges and reduce reliance on foreign support (9).

We present a set of indicators that would support the design and assessment of global health education initiatives that foster global justice and equity.

## 2 Main aspects of global health education justice

**Figure 1:**
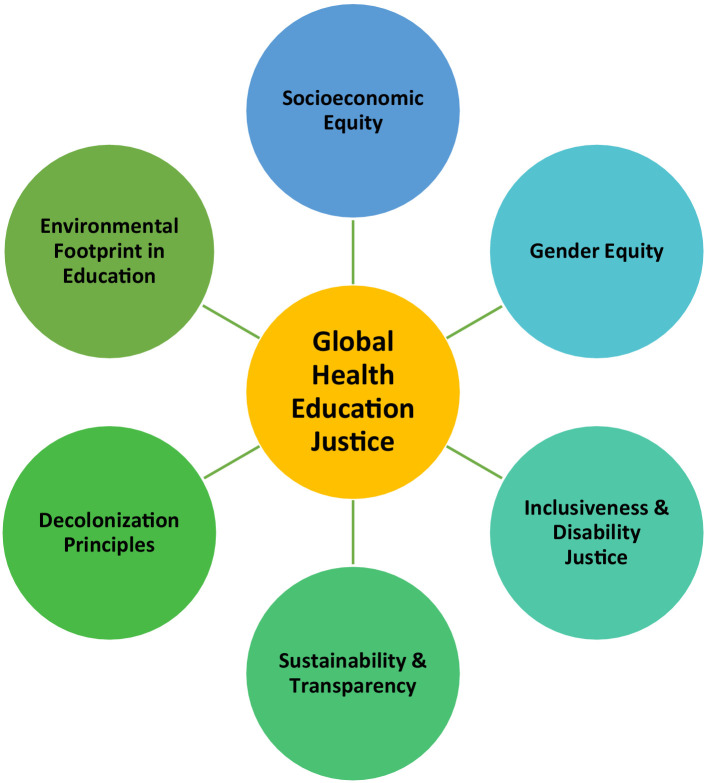
Main aspects of global health education justice.

We identified six main aspects of global health education justice: socioeconomic equity, gender equity, inclusiveness and disability justice, decolonization principles, sustainability and transparency, and environmental footprint in education (Figure 1). This classification framework was designed to categorize the key themes identified within the literature. We also propose a set of objectives and indicators (Supplementary Table 1) as a practical tool when designing, implementing, and evaluating GHEIs.

### 2.1 Socioeconomic equity

Socioeconomic factors strongly impact access to GHEIs for students from LMICs (16, 17). The financial aspect is the main barrier for motivated students who do not have enough funds to cover the costs associated with pursuing these opportunities abroad. Costs include, but are not limited to, travel, accommodation expenses, or visa application fees. Despite the availability of a scholarship, the total costs can still be overwhelming for early career PHP. The financial burden may be potentiated by employment issues, as pursuing educational programs often requires taking time off work or results in decreased income. Host institutions need to consider offering daily allowances to compensate for this work absence and take action to ensure that students do not have to choose between their professional responsibilities and their educational advancement.

Logistical barriers are very important for many international students, especially those who are unfamiliar with the environment of the host institution’s country (18). Some students find it difficult to make suitable travel and accommodation arrangements without guidance and support, while stress and uncertainty may discourage them from applying. Students who are parents also face an increase in childcare expenses, further complicating their participation in GHEI (19). Addressing these additional childcare costs can help decrease the financial burden on students leaving their home countries.

A way to acknowledge and address potential socioeconomic barriers is to assess each application individually (20). Host institutions need to rethink the design and delivery of GHEIs to ensure accessibility and inclusiveness. By creating meaningful support that takes into account the socioeconomic background of students from LMICs, organizations can empower a more diverse group of PHPs and ensure more just GHEIs.

### 2.2 Gender equity

The gender balance and diversity of students and lecturers is a step toward an inclusive learning environment. Host institutions should strongly promote gender equity in GHEIs, especially for students from LMICs. Strong representation of women and minority voices in GHEIs enhances the discourse by incorporating diverse perspectives and experiences (21, 22). Host institutions should strive for a gender-sensitive curriculum whenever possible, including topics such as reproductive health, gender-based violence, and the social determinants of health that disproportionately affect women and minorities.

Host institutions should keep in mind that cultural aspects and the prevalence of patriarchal norms in many LMICs significantly impact women’s access to education overall. To overcome these barriers, it is vital to empower women specifically to pursue global and public health education (23, 24). Host institutions should advocate supportive policies and enable GHEIs to encourage women’s participation and ensure they have sufficient resources to succeed. Host institutions can support students, particularly women and minorities, through mentorship opportunities to develop leadership skills and build professional networks. Networking events and leadership training should be financially supported if they are to lead to better gender-sensitive practices (21–24).

Implementing zero-violence policies in educational settings creates safe and inclusive student environments that are vital to effective learning. Such policies usually include implementing preventive measures regarding discrimination, hate speech, and all forms of harassment. To ensure the success of these actions within GHEIs, host institutions should establish clear strategic plans and determine responsibility.

### 2.3 Inclusiveness and disability justice

Numerous barriers discourage students with disabilities from pursuing educational opportunities, including GHEIs (25, 26). Inaccessible classrooms and other physical obstacles within host institutions often pose a challenge for students with mobility impairments. Similarly, the lack of alternative teaching materials formats can disengage students with sensory or cognitive disabilities. Without a responsible support person, students may struggle to advocate for their own needs, which adds to the emotional and logistical burden that can further discourage the participation of students with disabilities in GHEIs (25–27).

Achieving disability justice within GHEIs means that host institutions must actively address existing barriers, and physical accessibility is a fundamental first step. All teaching spaces must allow free movement for all GHEI participants. Host institutions are responsible for ensuring the accessibility of teaching materials and should provide online prerecordings, lecture transcripts, and closed captioning for videos. Full participation requires that students with disabilities are informed about these measures and can engage with course content however it suits them (25–28).

All participants should have access to information on the values of dignity and autonomy and training on creating a respectful and inclusive environment. GHEIs should empower students of all abilities and include diverse perspectives so that the future generation of PHPs can meet the needs of the entire population.

### 2.4 Decolonization principles

Neocolonial patterns continue to influence global health education and practice, often leading to dependency instead of promoting sustainable growth in LMICs (29, 30). Many global health interventions continuously rely on foreign expertise and resources, neglecting to invest in local capacity-building that empowers communities to achieve self-sustained improvements (6, 7). Without developing national public health infrastructure, GHEIs alone cannot cultivate a continuous supply of competent, locally trained PHPs capable of addressing the unique health challenges they face in their countries. Consequently, the long-term impact of GHEIs is limited, and LMICs often remain dependent on external support. Therefore, ensuring high-quality and accessible public health education within LMICs is essential for building autonomous and resilient public health systems (29–32). Considerable efforts have been made to identify the essential competencies required for the global public health workforce (33).

Host institutions are responsible for addressing these disparities, prioritizing the meaningful involvement of lecturers and students from LMICs. Additionally, many HIC-based host institutions encourage or require their students to engage in field experiences in LMIC communities for observation or project-based learning. While valuable, these experiences must adhere to ethical standards to prevent reinforcing power imbalances or burdening local communities without reciprocal benefits. Periodically, host institutions should consider relocating GHEI delivery to LMIC settings, even if primarily based in a high-income country. If they partner meaningfully with local education systems, host institutions can help strengthen the local supply of competent PHPs and promote a fair exchange of knowledge, skills, and resources (31, 32). This involves utilizing the existing capacity and lessons learned in the European region to assist other parts of the world (34).

Although English is often regarded as today’s *lingua franca*, host institutions should offer resources and educational materials in multiple languages whenever possible. Digital copies of materials that cannot be translated in advance should be made available to facilitate translation using accessible software. This approach ensures that language does not exclude the participation of students with limited English proficiency (35).

Incorporating decolonization principles into the curriculum is vital, even when the central theme is not directly related to global injustice. Host institutions should provide enough time for open discussions about decolonization and allow students to explore ways for global health justice promotion. GHEIs could raise awareness of existing power dynamics by integrating decolonization principles into their agendas.

### 2.5 Sustainability and transparency

Sustainability and transparency are core principles when designing and implementing GHEIs and ensuring GHEIs are ethically grounded (36–38). Host institutions should disclose any affiliations, partnerships, or financial interests related to GHEIs and provide complete transparency regarding their funding sources. Ethical financing means that host institutions avoid accepting support from industries that compromise the objectivity and integrity of GHEIs, such as tobacco, fossil fuels, high-level sugar drinks, or often pharmaceuticals (39). Maintaining such a strict policy against biased funding builds trust and ensures GHEI benefits global health justice (40).

A detailed agenda should be communicated well in advance because this ensures a productive learning experience and allows time for feedback and adjustments based on participants’ needs. The agenda should address pressing global health challenges and seek relevance (36, 38, 40).

Before designing GHEIs, host institutions should establish clear and measurable outcomes in consultation with LMIC colleagues. GHEI design needs to emphasize practical skill development and meaningful interaction whenever possible. GHEIs should also serve as a platform to enhance professional and peer networks, as these connections often translate to collaboration and support overall progress in knowledge. GHEIs prioritizing sustainability and transparency also promote more equitable and ethical practices in global health. With a set of clear objectives, host institutions can encourage meaningful, long-lasting collaborations among students and lecturers involved. Examples include collaborative research projects important for LMICs, co-developing educational programs that address local needs, and organizing joint public health interventions to build capacity and confront immediate challenges. Additionally, networks for young professionals and alumni can facilitate ongoing mentorship, knowledge exchange, and partnership opportunities.

### 2.6 Environmental footprint in education

Organizing any event with international participation has an environmental impact due to emissions from travel, food, materials, and waste production (41, 42). Host institutions should evaluate the environmental footprint of GHEIs, assessing carbon emissions and all other impacts of the event (43). Informing participants and the public about these carbon footprint estimates can raise awareness and highlight the institution’s commitment to environmental protection.

The host institution’s environmental footprint mitigation plan should outline practical steps to reduce its environmental impact. The use of printed materials, textiles, and plastics in welcome packages should be avoided, and reusable alternatives should be promoted whenever possible. Opting for digital access to all teaching materials is a part of sustainable practices and helps minimize waste (41–43).

Online participation is an essential part of the sustainable event model. Virtual participation should be made equal in terms of learning, networking, and other opportunities if host institutions are to minimize travel-related emissions while maintaining high-quality engagement. Inclusive online options promote a broader and more diverse range of participation, creating a globally connected learning environment with a lower environmental impact (44). Host institutions must identify and address potential barriers to online participation, especially those prevalent in low-income communities (power shortages, slow and unstable internet connection, lack of necessary equipment), which are often similar to challenges faced in telemedicine implementation.

Host institutions should promote sustainable transportation, accommodations, and food choices for all students and lecturers. Some of these actions are related to eco-friendly accommodation options, encouraging public transportation, and providing plant-based meals and sustainably sourced food. It is possible for GHEIs to foster a culture of sustainability among participants, aligning main GHEI outcomes with the broader goal of mitigating climate change (43, 44).

## 3 Discussion

Socioeconomic equity and decolonization in global health education are often approached by categorizing participants and lecturers according to the World Bank’s country classification system by income level. This method aims to balance representation between high-income countries (HICs) and low- and middle-income countries (LMICs). However, it frequently overlooks significant socioeconomic disparities within individual countries, necessitating assessments on a case-by-case basis. For example, a Global Health Summer School held in a HIC within the European Union (EU) may still be inaccessible to students from neighboring non-EU upper-middle-income countries or economically disadvantaged countries within the EU itself. This limitation arises because the World Bank classification relies solely on Gross National Income (GNI) per capita, which averages national income without considering internal inequalities. The relevance and impact of this classification in the global health context have been continuously criticized (45, 46).

Gender equity, disability justice, and inclusiveness have received increasing attention in global health education initiatives (GHEIs) in recent years, resulting in notable progress in discourse and visibility. Efforts have focused on promoting diversity in the global and public health workforce and advocating for equitable representation. For instance, Women in Global Health, a non-profit organization founded in 2015, champions equal representation in leadership, calls for a new social contract for women’s health and care workers, and promotes gender equity in global health (47). Similarly, international health authorities, including the WHO and regional associations of public health schools, have emphasized the importance of LGBT+ health by establishing dedicated working groups to address these issues. Additionally, initiatives have aimed to make GHEIs more inclusive for individuals with motor or cognitive disabilities by improving accessibility measures. However, the Global Health 50/50 Report from 2020, which evaluated 200 global organizations in health and health policy, revealed that over 70% of leaders are men, 80% are nationals of high-income countries, and 90% received their education in high-income countries. Meanwhile, women make up 70% of the health workforce but hold only 5% of leadership positions, highlighting ongoing gender inequities in global health leadership (48).

Sustainability, transparency, and the environmental impact of education are now key areas of focus within the global health movement. Concerns have intensified regarding the influence of industries associated with harmful health and environmental outcomes, such as tobacco, alcohol, processed foods, and fossil fuels (49). A notable example of this is the World Health Organization’s (WHO) recruitment policy, which disqualifies smokers from employment opportunities—a clear stance against the tobacco industry’s influence. However, progress in addressing other commercial determinants of health has been slower. Efforts to mitigate the impact of alcohol consumption, unhealthy diets fueled by processed foods, and carbon emissions from global health actions have given limited results. Additionally, many global health initiatives rely heavily on philanthropic funding, often from sources connected to systemic global health problems (50). Examining the flow of capital may reveal links to root causes like environmental destruction, extractivism, and unethical practices, creating a paradox within global health financing. This reliance raises important questions about the sustainability and ethical alignment of these initiatives, highlighting the need for greater accountability in global health education funding mechanisms.

## 4 Conclusion

Many regions of the world, especially low—and middle-income countries (LMICs), lack the capacity and competent workforce needed to build resilient public health systems that can effectively address emerging global threats. Substantial investments have been made in various global health education initiatives (GHEIs) to address these issues. However, ensuring justice in GHEIs is a complex task, often challenged by systemic barriers that can unintentionally perpetuate inequities and prevent disadvantaged persons from fully participating. These barriers include representation, financial and logistical constraints, and biases in the curriculum and program delivery. We propose a set of indicators to help host institutions identify and overcome these barriers during the design, implementation, and evaluation of GHEIs. By adopting a structured and reflective approach, host institutions can contribute to global health training that prioritizes equity, sustainability, and meaningful participation. Actions in this direction can strengthen the position of global health education as a powerful tool for achieving global health justice.
