# Supplementary material for: Ensuring justice in global health education initiatives – a review of pearls and pitfalls
Source: Front Public Health. 2025 May 27;13:1574917. doi: 10.3389/fpubh.2025.1574917 (PMC12184537; doi:10.3389/fpubh.2025.1574917)
Supplement: Supplementary file 1 [file Data_Sheet_1.pdf]

Table 1. A Checklist For Justice In Global Health Education Initiatives

ASPECT	COMPONENT	INDICATOR
**SOCIOECONOMIC EQUITY**	***Comprehensive Financial Support***	• Scholarships cover all essential costs: travel, accommodation, visa fees, and health insurance;<br>• Daily allowance (*per diem*) provided to cover meals, local transport, and incidental expenses;<br>• Transparent financial aid application process with clear criteria and accessible deadlines;
	Logistical Support	• Application assistance provided, including guidance on completing forms and submitting documentation;<br>• Visa application support with requirements and coverage of associated costs;<br>• Assistance with travel and accommodation arrangements, including local recommendations and resources;<br>• Welcome package with guidelines, cultural orientation, and local area recommendations;
	Childcare support	• Financial assistance or access to childcare facilities for participants with dependents;<br>• Childcare support information included in event materials and provided as part of financial aid options;
**GENDER EQUITY**	***Gender balance***	• Balanced representation of male and female participants, lecturers, and speakers;<br>• Targeted recruitment strategies to promote female participation and leadership roles;
	Gender-Sensitive Curriculum	• Event curriculum incorporates gender-specific issues, including women's health and diverse gender perspectives;
	Leadership Development	• Mentorship and leadership training opportunities tailored to the needs of both female and male participants;<br>• Leadership sessions structured to support inclusive professional development;

	Zero-Violence Policy	• Enforcement of a zero-tolerance policy against discrimination, harassment, and hate speech;<br>• Clear guidelines on Zero-Violence Policy communicated to all participants and personnel;
**INCLUSIVENESS & DISABILITY JUSTICE**	***Accessibility of Facilities***	• Full physical accessibility for classrooms, event venues, and teaching spaces;<br>• Hybrid participation options for those unable to attend in person due to health conditions;
	Accessible Learning Materials	• Provision of accessible materials, such as lecture transcripts, closed captioning, and recorded sessions;<br>• Additional support available for neurodivergent students, ensuring equitable learning opportunities;
	Diversity and inclusion	• Dedicated staff member to support the needs of students from diverse and underrepresented backgrounds.<br>• Pronouns of participants are respected and displayed prominently to foster inclusivity;
	Awareness and Training	• Distribution of guidelines promoting disability inclusive practices available to all attendees and facilitators;<br>• Designated person to monitor and uphold disability justice standards throughout the event;
**DECOLONIZATION PRINCIPLES**	***Representation of LMIC Participants***	• Active representation from all WHO regions, particularly among LMIC participants and lecturers;<br>• Collaborative engagement with LMIC institutions to foster mutual learning and fair knowledge distribution;
	Language Accessibility	• Simultaneous translation is available for non-English speakers when required;<br>• Materials provided in multiple languages, with digital copies for ease of access and translation;
	Decolonized Curriculum	• Curriculum addresses decolonization principles and global justice topics;<br>• Sufficient time allocated for discussions on these topics, encouraging critical engagement;

**SUSTAINABILITY & TRANSPARENCY**	***Theory of Change***	• Detailed report on the event's goals, theory of change, and intended impacts shared with participants beforehand;
	Outcome Reporting	• Publicly accessible report with clear outcomes, achieved milestones, and key lessons from the event;
	Achieved Relevance	• Participants have been included in discussions on curriculum design and provided feedback on their needs;
	Ethical Financing	• Financial transparency with funding sources declared, avoiding commercial interests that may bias content;<br>• Regularly published financial reports to maintain accountability;
	Participant Feedback	• Channels for participants to submit feedback, with transparent follow-up and response processes;
**ENVIRONMENTAL IMPACT OF EDUCATION**	***Carbon Footprint Management***	• Calculation and communication of the event's total carbon footprint to participants;<br>• Estimated individual carbon contributions shared with attendees;
	Environmental Mitigation Strategy	• Appointed coordinator to manage environmental impact reduction strategies;<br>• Public report on actions taken to minimize the environmental impact of the event;
	Digital Participation Options	• Meaningful online participation options to reduce travel-related emissions;<br>• Strategies in place to encourage virtual participation when appropriate;
	Sustainable Food Choices	• Fairtrade and sustainably sourced food and refreshments provided;<br>• Option for plant-based meals to reduce the carbon footprint of event catering;
